# Impacts of the Oxygen Precursor on the Interfacial Properties of La_x_Al_y_O Films Grown by Atomic Layer Deposition on Ge

**DOI:** 10.3390/ma10080856

**Published:** 2017-07-26

**Authors:** Lu Zhao, Hongxia Liu, Xing Wang, Yongte Wang, Shulong Wang

**Affiliations:** Key Laboratory for Wide Band Gap Semiconductor Materials and Devices of Education, School of Microelectronics, Xidian University, Xi’an 710071, China; lzhaoxd@163.com (L.Z.); xwangxd@163.com (X.W.); mikewyt@163.com (Y.W.); slwang@xidian.edu.cn (S.W.)

**Keywords:** atomic layer deposition, interfacial properties, La_x_Al_y_O, band alignments

## Abstract

Amorphous La_x_Al_y_O films were grown on n-type Ge substrate by atomic layer deposition using O_3_ and H_2_O as oxidant, respectively. A comparison of the XPS results indicated that a thicker interfacial layer with the component of LaGeO_x_ and GeO_x_ was formed at O_3_-based La_x_Al_y_O/Ge interface, causing lower band gap value as well as the conduction band offset (CBO) value relative to Ge substrate for O_3_-based La_x_Al_y_O film, with a concomitant degeneration in the interfacial properties. In contrast, for the H_2_O-based film, the leakage current of more than one order of magnitude less than that of O_3_-based La_x_Al_y_O film was obtained. All the results indicated that H_2_O is a more appropriate oxidant for improving the interfacial properties in the atomic-layer-deposited La_x_Al_y_O dielectric on Ge.

## 1. Introduction

With Si-based complementary-metal-oxide-semiconductor (CMOS) devices approaching their fundamental limits, high dielectric constant (high-k) materials grown on germanium and other high mobility semiconductors have been investigated to increase the drain current in the channel region [1,2]. Unfortunately, one primary challenge for Ge used in MOSFET devices is generally the poor electrical performance of native Ge oxide, resulting in poor interfacial properties at the insulator/Ge interface for most high-k dielectrics deposited on Ge substrate without any surface passivation process [3,4]. In order to improve the interface quality, appropriate passivation should be carried out. Attentions had been focused on the formation of thermally grown GeO_2_ prior to the high-k dielectrics deposition process [5,6]. However, GeO_2_ becomes unstable at high temperature when deposited on Ge because it would react with Ge atoms to form substoichiometric oxide or volatile GeO [7,8], deteriorating the electrical performance of Ge-based MOS devices. Recently, rare earth oxides have been considered as a promising passivation interlayer for high-k dielectric grown on Ge [9]. Furthermore, La-based dielectric materials have been shown to form a good passivation layer due to the formation of a stable La germanate compound on Ge substrate which could prevent the formation of volatile GeO [10,11]. Among various deposition methods for growing high-k dielectric films, atomic layer deposition (ALD) has been considered as one of the most promising technique to produce high-k dielectric films in high quality due to the outstanding characteristics for precise thickness and composition control, excellent uniformity and process compatibility to conventional CMOS process [12,13]. O_3_ and H_2_O are two kinds of oxygen source precursors commonly used in the ALD process. It has been reported that the difference of oxidants would have an impact on the ALD reaction mechanism and surface chemistry of the deposited film [14], with a further influence on the relative electrical properties.

In this paper, the effect of H_2_O and O_3_ on the interfacial properties of La_x_Al_y_O films grown by atomic layer deposition on Ge was systematically investigated. X-ray photoelectron spectroscopy (XPS) analysis of the LaAlO_3_ films was used to provide direct observation on the band alignments of La_x_Al_y_O films relative to Ge substrate. Attentions were focused on the electrical performance of La_x_Al_y_O/Ge structures to analyze the influence of different oxygen precursors on the interfacial properties.

## 2. Experiment

La_x_Al_y_O gate dielectric films were deposited on n-type Ge (100) wafers with resistivity of 0.1–1 Ω·cm by ALD technique using La(^i−^PrCp)_3_ and TMA as La and Al precursor, while two kinds of oxygen source precursors (O_3_ and H_2_O) were used as oxidant, respectively. Prior to the deposition, Ge substrates were treated with acetone and hydrous alcohol, and then dipped into 2%-HF solution for 30 s to remove the native GeO_x_ layer, followed by a 60 s rinse in de-ionized water. The precursors were alternately introduced to the reactor chamber using high purity N_2_ (>99.999%) as the carrier gas. A typical ALD growth cycle for La_2_O_3_ with O_3_ used as the oxygen precursor was 0.1 s La(^i−^PrCp)_3_ pulse/4 s N_2_ purge/0.3 s O_3_ pulse/10 s N_2_ purge, whereas for Al_2_O_3_ with O_3_ used as the oxygen precursor, it was 0.1 s TMA pulse/3 s N_2_ purge/0.5 s O_3_ pulse/4 s N_2_ purge. Moreover, when H_2_O was used as the oxygen precursor, a typical ALD growth cycle for La_2_O_3_ was set as 0.3 s La(^i−^PrCp)_3_ pulse/4 s N_2_ purge/ 0.3 s H_2_O pulse/9 s N_2_ purge, while for Al_2_O_3_, it was 0.1 s TMA pulse/3 s N_2_ purge/0.1 s H_2_O pulse/4 s N_2_ purge. Using these process parameters, for La_2_O_3_, a linear relation with a growth rate of approximately 0.85 Å/cycle was obtained, and the steady-state growth rate of Al_2_O_3_ films was approximately 0.93 Å/cycle with O_3_ as the oxygen precursor. Besides, when H_2_O was used as the oxidant, the growth thickness per ALD cycle for La_2_O_3_ was ~0.75 Å, while the growth rate of Al_2_O_3_ was approximately 0.92 Å/cycle. At the deposition temperature of 300 °C, the film thickness was tuned to fix at ~10 nm and ~5 nm by varying the number of ALD cycles while setting the La/Al pulse ratio as 1:1. 

Post-deposition rapid thermal annealing was carried out at 600 °C for 90 s in N_2_ ambient. The crystallization characteristics of the La_x_Al_y_O films were checked by grazing incidence X-ray Diffraction (GIXRD) at the angle of incidence ω = 0.5°. None of the films reveal any diffraction peaks, indicating in the thermal stability of the films. The physical thickness of the deposited films was optically measured using Woollam M2000U (Woollam Co. Inc., Lincoln, NE, USA) spectroscopic ellipsometry (SE) by fitting the ellipsometry data using a Gen-Osc mode consisting of Gaussian and Tauc-Lorentz oscillators and considering the native GeO_x_. The composition and band structure of the deposited La_x_Al_y_O films was examined by XPS measurements. All the wafers were etched by Ar^+^ ion beam bombardment for 10 s (~0.26 nm/s) to remove the influence of the impurities on the surface. C 1*s* peak from adventitious carbon at 284.6 eV was used as an internal energy reference during the XPS analysis. In this experiment, the ~10 nm La_x_Al_y_O film was used to obtain the XPS spectra for thick amorphous La_x_Al_y_O, and the ~5 nm La_x_Al_y_O/Ge structure was thin enough to obtain XPS spectra from both the La_x_Al_y_O film and the underlying germanium substrate. The electrical properties of the 5 nm films were measured using a metal-insulator-semiconductor (MIS) capacitor structure. A metal gate with a diameter of 300 μm was fabricated by depositing 150 nm Al using the electron-beam evaporation through a shadow mask, followed by annealing in forming gas ambient (97% N_2_/3% H_2_) at 400 °C for 20 min. The capacitance-voltage (*C*-*V*) and leakage current density-voltage (*J*-*V*) measurements were carried out using Agilent B1500A analyzer.

## 3. Results and Discussion

**Figure 1:**
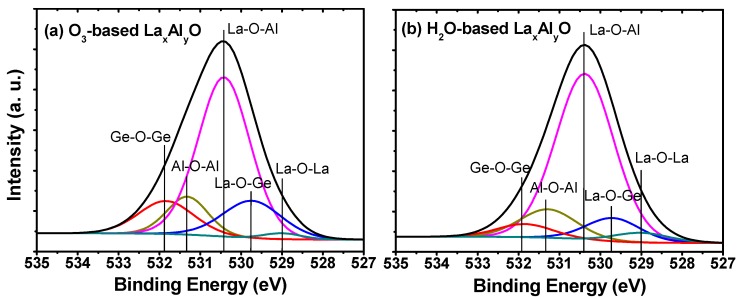
Shallow core-level spectra of O 1*s* for the 5 nm (**a**) O_3_-based and (**b**) H_2_O-based La_x_Al_y_O films.

As shown in Figure 1, the variations in O 1*s* XPS spectra for the 5 nm O_3_-based and H_2_O-based La_x_Al_y_O films annealed at 600 °C were analyzed to investigate the chemical bonding states near the La_x_Al_y_O film and Ge substrate interfaces. The O 1*s* spectra were fitted with five Gaussian–Lorentzian line-shaped peaks, which are at 529.0, 529.7, 530.4, 531.3 and 531.9 eV. These peaks correspond to the chemical bonds of La-O-La, La-O-Ge, La-O-Al, Al-O-Al and Ge-O-Ge, respectively [15,16,17]. For La-O-La, La-O-Al and Al-O-Al chemical bonds; the intensity of the peaks varies slightly, indicating the difference of oxidant has negligible influence on the chemical bond structures of the upper deposited La_x_Al_y_O layers. However, compared with the La_x_Al_y_O film using H_2_O as oxidant, an obvious increment in the intensity of La-O-Ge and Ge-O-Ge peaks could be observed for the O_3_-based La_x_Al_y_O film, which illustrates that more interfacial oxide layer (mainly consisting of LaGeO_x_ and GeO_x_) was formed at the O_3_-based La_x_Al_y_O/Ge interface during the deposition and post-deposition annealing process [18], which may be caused by the higher oxidability of O_3_ [19].

**Figure 2:**
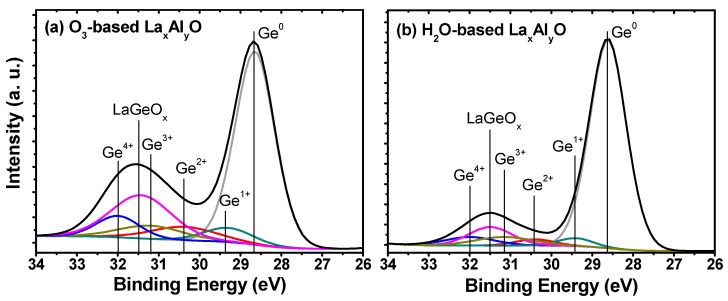
Shallow core-level spectra of Ge 3*d* for the 5 nm (**a**) O_3_-based and (**b**) H_2_O-based La_x_Al_y_O films.

In order to study the chemical bonding states near the La_x_Al_y_O film and Ge substrate interfaces more clearly, further investigation was applied to the variations in Ge 3*d* XPS spectra for the 5 nm O_3_-based and H_2_O-based La_x_Al_y_O films, as shown in Figure 2. The Ge oxide (GeO_x_) spectra, which are located at a higher binding energy with respect to the Ge^0^ peak originating from the Ge substrate, can be deconvoluted into four GeO_x_ peaks (Ge^1+^, Ge^2+^, Ge^3+^, Ge^4+^) with energy shift of 0.8, 1.8, 2.6, and 3.4 eV, respectively. These GeO_x_ species were likely present due to the formation of an interfacial layer between the La_x_Al_y_O film and Ge substrate. Here, the Ge^4+^ peak originates from GeO_2_, and other Ge^1+^, Ge^2+^ and Ge^3+^ peaks originate from Ge sub-oxides [20]. A comparison of Figure 2a,b revealed the same variation trend of the formation of interfacial oxide layer as analysed in the O 1*s* XPS spectra; that is, larger amounts of LaGeO_x_ and GeO_x_, including GeO_2_ and Ge sub-oxides, were formed at La_x_Al_y_O/Ge interface in the O_3_-based case. The variation of these interfacial oxides would have an influence on the interfacial characteristics of La_x_Al_y_O film/Ge structure and then affect its electrical properties, and this aspect will be discussed in detail later in this paper.

**Figure 3:**
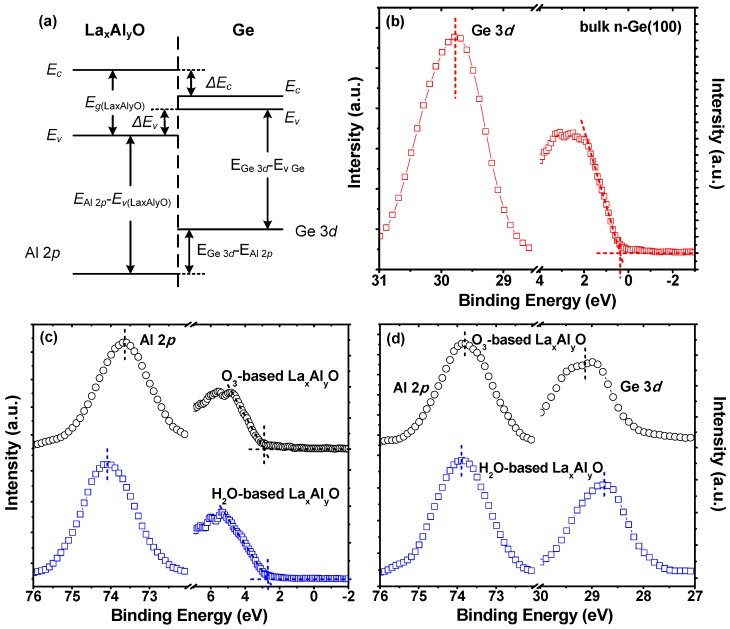
(**a**) schematic of band energy alignment diagram for a La_x_Al_y_O/Ge structure; XPS core level spectra of (**b**) Ge 3*d* and valence band for bulk clean n-Ge(100); (**c**) Al 2*p* and valence band for 10 nm La_x_Al_y_O films; and (**d**) Al 2*p* and Ge 3*d* for 5 nm La_x_Al_y_O films on n-Ge(100).

The band offsets of La_x_Al_y_O films relative to the Ge substrate were determined by a core level photoemission-based method similar to that of Kraut et al. [21,22], as illustrated in Figure 3a. Accordingly, the valence band offset (VBO, Δ*E*_v_) is given by Equation (1):

(1) $$\Delta E_{v}={\left(E_{\mathrm{Ge}\;3d}-E_{V}\right)}_{\mathrm{Ge}}-{\left(E_{\mathrm{Al}\;2p}-E_{V}\right)}_{{\mathrm{Thick}\;\mathrm{La}}_{x}{\mathrm{Al}}_{y}O}-{\left(E_{\mathrm{Ge}\;3d}-E_{\mathrm{Al}\;2p}\right)}_{{\mathrm{La}}_{x}{\mathrm{Al}}_{y}O/\mathrm{Ge}}$$

where ${\left(E_{\mathrm{Ge}\;3d}-E_{V}\right)}_{\mathrm{Ge}}$ is the energy difference between Ge 3*d* and valence band maximum (VBM) in the bulk clean Ge substrate, as shown in Figure 3b; ${\left(E_{\mathrm{Al}\;2p}-E_{V}\right)}_{{\mathrm{Thick}\;\mathrm{La}}_{x}{\mathrm{Al}}_{y}O}$ is the energy difference between Al 2*p* and VBM in the 10 nm La_x_Al_y_O film, as shown in Figure 3c; and ${\left(E_{\mathrm{Ge}\;3d}-E_{\mathrm{Al}\;2p}\right)}_{{\mathrm{La}}_{x}{\mathrm{Al}}_{y}O/\mathrm{Ge}}$ is the energy difference between Ge 3*d* and Al 2*p* core levels in the 5 nm La_x_Al_y_O on n-Ge(100), as shown in Figure 3d. Then, according to Equation (1), the VBOs for the films with O_3_ and H_2_O as oxidant can be figured out as 3.34 and 3.11 eV, respectively.

The corresponding conduction band offset (CBO, Δ*E*_c_) between La_x_Al_y_O and Ge can be obtained by Equation (2):

(2) $$\Delta E_{c}=E_{g}{}_{\left({\mathrm{La}}_{x}{\mathrm{Al}}_{y}O\right)}-\Delta E_{v}-E_{g}{}_{\left(\mathrm{Ge}\right)}$$

It is generally known that the band gap of germanium is 0.67 eV at room temperature. In order to obtain the CBOs of La_x_Al_y_O films relative to germanium, the band gap of amorphous La_x_Al_y_O on Ge substrate needs to be determined.

**Figure 4:**
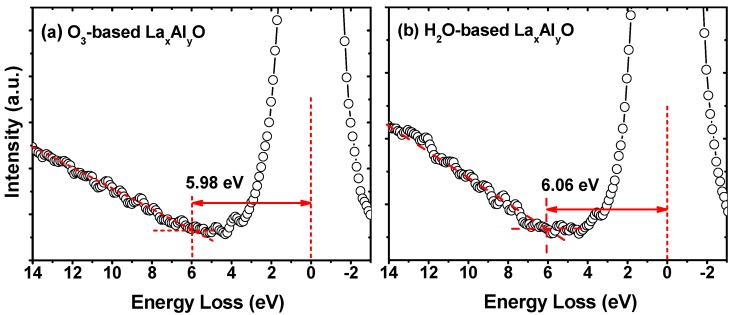
O 1*s* energy-loss spectra for the 10 nm (**a**) O_3_-based and (**b**) H_2_O-based La_x_Al_y_O films.

The band gaps of La_x_Al_y_O films were measured by examining the energy loss of the O 1*s* core levels for the 10 nm samples by XPS measurements. After being etched for ~2 nm, the XPS spectra signals can be considered as coming from the pure deposited films. In principle, the photoexcited electrons passing through dielectric films can suffer inelastic losses due to plasmon (collective oscillation) and single particle excitation (band-to-band transition excitation) [23]. It is proved that the band gap equals the energy distance between the photoemission peak centroid and the onset of the features due to single particle excitations, and it is usually obtained from the inelastic energy loss features observed on the high binding energy side of the core level photoemission peaks [24]. Besides, the onset of the O 1*s* loss spectrum can be determined by linearly extrapolating the segment of maximum negative slope to the background level [25,26]. Using this method, as shown in Figure 4, the band gaps of the O_3_-based and H_2_O-based La_x_Al_y_O films were determined to be 5.98 and 6.06 eV, respectively. Accordingly, the CBOs of O_3_-based and H_2_O-based La_x_Al_y_O films relative to Ge were figured out as 1.97 and 2.28 eV, respectively.

**Figure 5:**
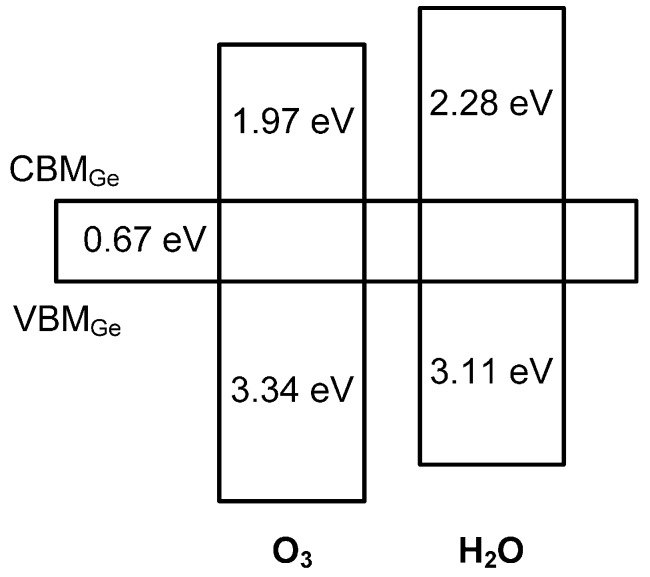
Results of the calculated band offsets for the La_x_Al_y_O/Ge structures with O_3_ and H_2_O as oxidant.

Results of the calculated band gaps and band offsets are shown in the schematic diagram in Figure 5. It is worth noting that the band gap values of the deposited La_x_Al_y_O films are smaller than those of pure amorphous La_x_Al_y_O film of ~6.2 eV [27], which implies that the composition of the deposited film is not pure La_x_Al_y_O. As is known; to some extent, the influence of the XPS signals from the possible interfacial oxide layer (GeO_2_, *E_g_*~ 5.8 eV) would diminish the band gap values of the deposited La_x_Al_y_O films [28]. Thus, the variation of the band gaps would reflect the degree of the formation of interfacial oxide layer between the deposited La_x_Al_y_O film and Ge substrate. That is, a thicker interfacial oxide layer should exist at the O_3_-based La_x_Al_y_O/Ge interface, as the band gap of O_3_-based La_x_Al_y_O film is slightly smaller than that of the H_2_O-based sample. This result is in good agreement with the interfacial chemical bonds information extracted from the O 1*s* and Ge 3*d* spectra as mentioned above. In addition, the CBO of GeO_2_ relative to Ge (~0.54 eV) is much smaller than that of La_x_Al_y_O on Ge (~2.2 eV) [28,29]. Consequently, due to the existence of a thinner interfacial layer, a bigger value of CBO is obtained when H_2_O was used as oxidant.

**Figure 6:**
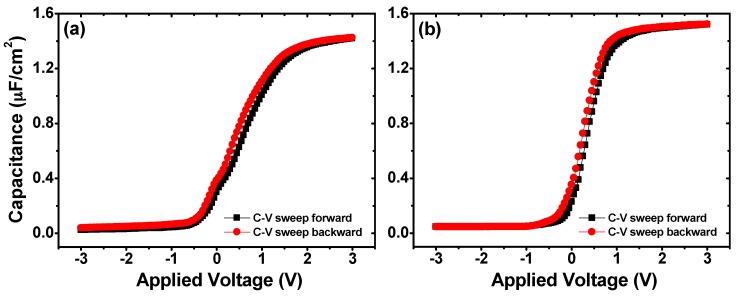
*C*-*V* characteristics of MIS capacitors using 5 nm (**a**) O_3_-based and (**b**) H_2_O-based La_x_Al_y_O films as insulators.

Figure 6 shows the *C*-*V* characteristics of the fabricated MIS capacitors using 5 nm O_3_-based and H_2_O-based La_x_Al_y_O films as insulators. For simplicity, the MIS capacitor structures using O_3_-based and H_2_O-based La_x_Al_y_O films as insulators were assigned as MIS capacitor S1 and MIS capacitor S2, respectively. The *C*-*V* curves were obtained by sweeping forward (bias from negative to positive) and backward (bias from positive to negative) at a frequency of 100 kHz. The flat band voltages (*V*_FB_) of the *C*-*V* curves were extracted from the simulation software Hauser NCSU CVC program, taking into account quantum mechanical effects [30]. Compared with MIS capacitor S2, a positive *V*_FB_ shift could be observed in the *C*-*V* curves for MIS capacitor S1, which is an indication of the presence of more effective negative oxide charges in the bulk of the O_3_-based gate dielectric. Ruling out the influence of generally positive charged fixed oxide charges (*Q*_f_) and mobile ionic charges (*Q*_m_), the oxide trapped charges (*Q*_ot_) negative charged were suspected to be responsible for the positive shift of *V*_FB_ [31]. The charge trapping behavior of the fabricated capacitors was investigated through the *C*-*V* hysteresis characteristics. The hysteresis width (Δ*V*_FB_) extracted from the dual-swept *C*-*V* curves for MIS capacitors S1 and S2 are 154 and 95 mV, respectively. For the O_3_-based sample, a larger Δ*V*_FB_ of the dual-swept *C*-*V* curves illustrates the existence of more oxide trapped charges in the O_3_-based gate dielectric, which is in consistent with the shift tendency of *V*_FB_. Additionally, it is worth noting that, compared with what is shown in Figure 6b, the *C*-*V* curves for MIS capacitor S1 (Figure 6a) slope gently and exhibit a more obvious anomalous hump phenomenon in the weak inversion region, indicating the formation of more interface traps at the O_3_-based La_x_Al_y_O film/Ge interface.

From the XPS results as mentioned above, we can conclude that a thicker interfacial layer consisting of LaGeO_x_ and GeO_x_ exists between O_3_-based La_x_Al_y_O film and Ge substrate. Such an interfacial layer, as reported, has a much lower dielectric constant (5~6) than that of La_x_Al_y_O [32,33], resulting in a smaller accumulation capacitance value for MIS capacitor S1. Being a thermally stable germanate compound on the surface of Ge substrate, LaGeO_x_ was reported to be of help in suppressing Ge out-diffusion and improving interface quality. However, among the germanium oxides, GeO is volatile and sublimes leaving behind a defective interface contained lots of defects and dangling bonds, which makes it known to have an adverse influence on the interfacial properties [11]. Additionally, it has been reported that at temperatures of up to 430 °C, GeO_2_ becomes unstable, and will react with substrate Ge atoms generating volatile GeO, following the reaction of GeO_2_ + Ge → 2GeO [7]. Therefore, compared with the H_2_O-based La_x_Al_y_O, the increase in oxide-trapped charges and interface traps in O_3_-based La_x_Al_y_O film/Ge structures should be attributed to the extra formation of volatile GeO.

**Figure 7:**
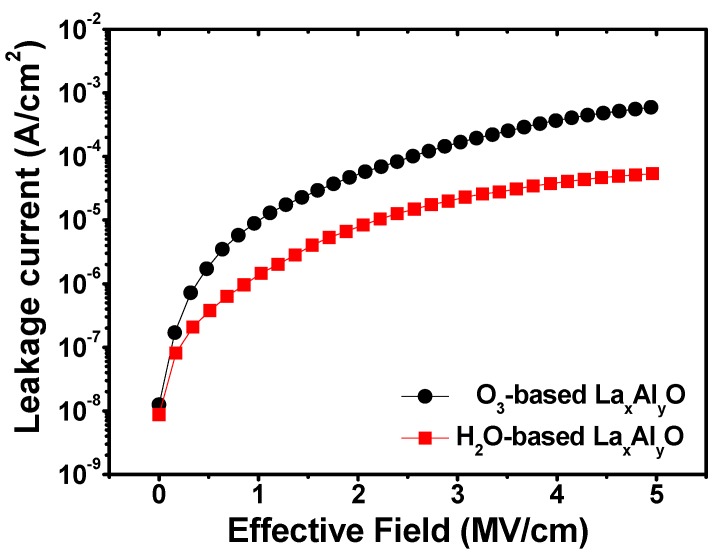
*J*-*V* characteristics of MIS capacitors using 5 nm O_3_-based and H_2_O-based La_x_Al_y_O films as insulators.

Figure 7 shows the leakage current density as a function of the applied electrical field for the fabricated Al/5 nm La_x_Al_y_O/n-type Ge capacitor structure. As we know, the polarity of gate leakage current through gate dielectrics depends on the gate bias polarity and substrate doping type. For the n-type Ge substrate used in this work, electron injection from the conduction band is the dominant tunneling current component under positive gate bias [34]. At the applied electrical field of 3 MV/cm, the leakage current density of the O_3_-based and H_2_O-based film was measured to be 2.29 × 10^−5^ and 1.68 × 10^−4^ A/cm^2^, separately. Compared with the O_3_-based La_x_Al_y_O film, a decrease of more than one order of magnitude in the leakage current density was found for the H_2_O-based film. Such a decrease is suspected of benefiting from the larger conduction band offset mentioned above. The larger conduction band offset means the existence of higher potential barriers between the La_x_Al_y_O film and n-Ge substrate, which would weaken the tunneling effect of electrons in the MIS capacitors, resulting in lower gate leakage current. In addition, less structural defects and dangling bonds in the H_2_O-based La_x_Al_y_O film/Ge structure mean a smaller possibility to create a conduction path by forming a continuous chain connecting the gate to the semiconductor, which may also provide an explanation for the significant decrease of gate leakage current in MIS capacitors S2.

## 4. Conclusions

In this paper, amorphous La_x_Al_y_O films were deposited on Ge substrate by ALD using O_3_ and H_2_O as oxygen precursor, respectively. Due to the higher oxidability of O_3_, the formation of interfacial layer (mainly consisting of LaGeO_x_ and GeO_x_) was enhanced at O_3_-based La_x_Al_y_O/Ge interface, leading to a slight decrease of the band gap for O_3_-based La_x_Al_y_O film, as well as the CBO value relative to Ge substrate compared with that of the H_2_O-based sample. Additionally, the extra formation of volatile GeO causes the increase of oxide trapped charges and interface traps in O_3_-based La_x_Al_y_O film/Ge structure. As a result, a much lower gate leakage current was obtained when the H_2_O-based La_x_Al_y_O film was used as MIS gate insulator, indicating that H_2_O is a more appropriate oxidant applied for the deposition of La_x_Al_y_O dielectric on Ge substrate to achieve suitable band alignments and favorable interfacial properties.
